# Factors associated with non-use of modern contraceptives among sexually active women in Ethiopia: a multi-level mixed effect analysis of 2016 Ethiopian Demographic and Health Survey

**DOI:** 10.1186/s13690-022-00922-2

**Published:** 2022-07-06

**Authors:** Solomon Sisay Mulugeta, Setegn Muche Fenta, Kenaw Derebe Fentaw, Hailegebrael Birhan Biresaw

**Affiliations:** Department of Statistics, College of Natural and Computational Sciences, Debre Tabor University, Debra Tabor, Ethiopia

**Keywords:** EDHS, Ethiopia, Modern contraceptive, Multilevel logistic, Reproductive age, Women

## Abstract

**Background:**

Appropriate contraceptive use prevents unintended pregnancy, protects the health of mother and child, and promotes women’s well-being. Use of modern Family planning in Ethiopia was still very low. The purpose of this study was to assess the factors that are associated with non-use of modern family planning services among women of reproductive age.

**Method:**

A nationally representative 2016 EDHS women data were used for analysis. A total of 15,683 women in the reproductive age group were included in this study. Descriptive and multilevel multivariable binary logistic regression models were used to summarize descriptive data and measure statistical association between the dependent and the individual and community level variable, respectively. Adjusted Odds Ratio (AOR) and confidence interval were respectively used to measure association and its statistical significance.

**Result:**

Among women in the reproductive age group 79.49% (95% CI: 78.85%, 80.12%) did not use a modern contraceptive method. Women age between 25–34 years (AOR = 0.54, 95% CI: 0.47–0.61) and age between 34–49 year (AOR = 0.62, 95% CI: 0.55–0.71), having primary educated women (AOR = 0.0.77, 95% CI: 0.68–0.87),secondary and above educational (AOR = 0.88, CI: 0.75–1.03), Secondary and above-educated husband (AOR = 0.84, 95% CI: 0.72–0.96), rich women (AOR = 0.74,95%CI:0.65–0.85), health facility delivery (AOR = 0.84, 95%CI: 0.73–0.0.98), being watching TV (AOR = 0.74, 95% CI: 0.65–0.85), having 1–2 living children (AOR = 0.21, 95% CI: 0.19–0.23) are less likely to not use contraception were identified. Furthermore, Muslim women (AOR = 1.43, 95% CI: 1.23–1.62), women living in rural area (AOR = 3.43; 95% CI: 2.72–4.32), and ANC visit 1.25(1.07–1.47) were more likely to not use contraception. Further, Women in Afar, Somali, Gambela, Harari, and Dire Dawa were less likely to use modern contraception methods than women in Tigray, but Amhara region had a lower rate of non-use.

**Conclusion:**

Family planning interventions should target younger women, women living in rural areas, the poor, and Muslim women. Moreover, initiatives to empower women associated to family planning programs would be beneficial in increasing contraceptive uptake among sexually active women in Ethiopia.

## Background

In the world, 842 million women used modern contraception to avoid unintended and unwanted pregnancies. The world population had reached 7.7 billion people by mid-2019, up one billion since 2007 and two billion since 1994. The world population is expected to reach 8.5 billion in 2030, 9.7 billion in 2050, and 10.9 billion in 2100. Sub-Saharan Africa will account for the majority of global population growth in the coming decades, while many other regions will begin to see population decline. Between 2019 and 2050, the global population is expected to grow by almost two billion people, with 1.05 billion (52%) of that growth occurring in Sub-Saharan African countries [1]. Ethiopia is the most populous country in Sub-Saharan Africa, with a population of 112 million people and a fertility rate of 4.6 children per woman. Pregnancy risks are higher in Africa due to a high fertility rate, poor health condition, and a lack of access to medical care [2]. The health service's Family Health division is in charge of achieving the aims of the Family Planning Program (FPP), and several government and non-governmental organizations are aiding Family Health in achieving the government's national and international targets. The primary goal of the FPP is to increase access to high-quality health care, including FPP services, without causing financial hardship [3–5].

In Sub-Saharan Africa, 23% of married women use family planning, with 18% using a modern approach and 5% using a traditional one. However, a larger amount of women 25% report having a “unmet need”, which means they would like to stop having children or delay their next delivery, but are not adopting any kind of family planning [6].Unintended pregnancies and dangerous abortions can both be avoided with proper family planning [7, 8]. Despite their value, modern contraceptives are not universally available or used. In comparison to women in developing countries, developed-country women have better access to and use of modern contraceptives [9–11].

In Ethiopia, a common healthcare challenge is the use of contemporary contraception. Despite the fact that women are using contemporary contraception at a higher rate than ever before, there are still obstacles. Within the country, there are many differences in the use of contemporary contraceptives. In comparison to Addis Ababa, the Somali area has the lowest rate (1.4%) of contemporary contraception use (50.1%) [5, 12]. In Ethiopia, maternal mortality rates during pregnancy stood at 412 per 100,000 live births [12]. In 2017, Ethiopia had more maternal deaths than any other country in the world, reported at 14,000 deaths. Hence, Ethiopia has a huge task to achieve SDG4 & 5 by 2030 [13]. In Ethiopia, postpartum modern contraceptive use ranges from 12.05% to 80.32 percent [14, 15]. Women’s occupation, awareness of FP, discussion with husband, support from husband, age of women, parity, and household wealth status were found to be factors associated with contraceptive use [14, 16–18].

Despite the existence of numerous individual studies identifying the factors which contribute to modern contraceptive use among women in reproductive age groups, there is no single study that covers all regions in Ethiopia in terms of the prevalence and associated factors which contribute to non-use of modern FP among women in reproductive age groups [14, 16, 19–25]. Although the fact that those studies used a regression analysis that only looked at individual-level factors and ignored community effects (clusters). For this purpose, an ordinary logistic regression model can be used to make incorrect conclusions. The study’s enumeration area seems to have contributed to the variability in the factors associated with non-use of modern contraceptive. Due to this, we proposed a binary multilevel logistic regression model to assess non-use of modern contraception [26, 27]. As a result, the purpose of this study was to assess the factors that are associated with non-use of modern contraceptive utilization among women of reproductive age. As a result, this study will attempt to fill this gap, which may aid planners and policymakers in developing effective strategies to reduce the overwhelming complications of unintended pregnancy while also improving the national and regional socioeconomic status.

## Materials and methods

### Study design and setting

For analysis, data from the 2016 Ethiopian Demographic and Health Surveys were used. The EDHS 2016 survey was designed to provide estimates of key indicators for the entire country, for urban and rural areas separately, and for each of the nine regions and two administrative cities (Tigray, Afar, Amhara, Oromia, Somali, Benishangul-Gumuz, SNNPR, Gambela and Harari). Each region was divided into urban and rural areas, resulting in 21 different sample strata. In each stratum, samples of Enumeration Areas (EAs) were chosen in two stages. Based on the 2007 PHC, an independent selection was implemented in each sampling stratum, involving a total of 645 EAs (202 in urban and 443 in rural) areas with probability proportional to EA size in the first stage. The second stage involved selecting a fixed number of 28 households per cluster using an equal probability systematic selection from the newly created household listings [12].

### Source of data

The dataset used in this study was obtained from the MEASURE DHS database, which can be found at http://dhsprogram.com/data/ after receiving approval from the DHS program office for the 2016 Ethiopian Demographic and Health Survey, the fourth comprehensive survey.

### Study variables

#### Dependent variable

The dependent variable is current non-use of modern contraception among women of reproductive age. Women were classified as “non-users” if they did not use any modern contraceptive method or used Folkloric and Traditional methods, and “users” if they used any modern contraceptive method.

#### Independent variables

Previous research and the variable's availability in the 2016 EDHS dataset were used to determine the independent variables for non-use of modern contraceptive methods. Variables were divided into two categories: individual level variables and community level variables associated for a multilevel logistic approach.

#### Individual-level variables

Age of women at the time of survey, husband age, educational level of women, educational level of husband, women occupation, wealth index, husband’s occupation, marital status, religion, health care delivery, Accessing Health Care, desire for more children, births in the last 5 years, hearing of family planning messages through radio, Watched family planning on TV last few months, Read family planning in newspaper/magazine last few months, and ANC visit were included as individual-level variables.

#### Community –level variables

Region and place of residence were regarded as community-level variables obtained directly from EDHS, but the remaining variables were not obtained directly from EDHS.

### Data management and analysis

SPSS software version 23 was used to extract and decode data, and STATA version 14 was used to analyze the decoded data. To describe the study respondents, descriptive statistics such as frequencies, and percentages were used. A multilevel study design does not consider individual observations to be independent of one another. The women in this study are nested with Enumeration Areas (EAs). The standard regression model is inapplicable in this situation. As a result, a multilevel logistic regression model was used to identify the associated predictors of modern contraceptive non-use among sexually active women in Ethiopian.

Four consecutive models were fitted in the multilevel analysis [28, 29]. The first is the null model (Model I), which is fitted without any explanatory variable at the individual and community levels to detect the existence of a possible contextual effect. The second model fit by incorporating all individual-level data variables (model II). This step evaluates the contribution of each individual-level explanatory variable, the significance of each predictor, and the changes in the first- and second-level variance terms. The third model was created by incorporating all community-level variables (Model III). This model allows us to examine whether the explanatory variables at the community level explain the between-group variation in the dependent variable.

### Model building

We fit four models: the null model (no explanatory variables), model I (individual-level variables only), model II (community-level variables only), and model III (individual-level and community-level variables both). Since the models were nested, model comparison and fitness were based on the Intra-class Correlation Coefficient (ICC), Likelihood Ratio (LR) test, Median Odds Ratio (MOR), and deviance (-2LLR), Akaike’s Information Criteria (AIC), and Bayesian Information Criteria (BIC) values. As a result, model III (individual + community) was the best fit for this study [30, 31].

### Parameter estimation method

Fixed effect estimates in the multilevel multivariable logistic regression model measure the relationship between non-use of modern contraceptive method and individual and community level factors. To select eligible variables for multivariable analysis, bivariate analysis was performed. Variables with *P*-values ≤ 0.2 were eligible and chosen for the multivariable analysis [32]. In the multivariable analysis, the Adjusted Odds Ratio (AOR) with 95 percent CI was reported, and variables with a *p*-value ≤ 0.05 were considered a significant factor influencing non-use of modern contraceptive method. When selecting two clusters at random, non-use of contraceptive use was found in both.The random effect measures variation of non-use of modern contraceptive method across clusters expressed by Intra-class Correlation Coefficient (ICC) which measures the degree of heterogeneity of non-use of modern contraceptive method between clusters, Percentage Change in Variance (PCV) indicating the proportion of the total observed individual variation of non-use of modern contraceptive method that is attributable to between cluster variations, and Median Odds Ratio (MOR) which shows the median value of the odds ratio between the cluster at high non-use of modern contraceptive utilization and cluster at low non-use of modern contraceptive utilization, when randomly select out two clusters [28]. The variance inflation factor (VIF) test was used to check for multicollinearity, and all variables had a VIF of less than five and a tolerance of larger than 0.1, indicating that there was no multicollinearity [33].

## Result

### Background characteristics of women of reproductive age group

A total of 15,683 women in the reproductive age group were included in the analysis. Only 3217 (20.51%) of the 15,683 women in the reproductive age group were using a modern contraceptive method, while the remaining 12,466 (79.49%) did not use a modern contraceptive method. The majority of women (40.81%) are between the ages of 15 and 24 and 44.16% have not used a modern contraceptive method, while more than two-thirds of their husbands (76.02%) are between the ages of 34 and 59. The highest number of women were included from Oromia, 1892 (12.06%), of which 11.99% did not utilize modern contraception, while the lowest number of women were included from Harari, 906 (5.78%). Over three out of every five women, 10,335 (65.90%), were from rural areas, of this 67.23% not using modern contraception. The majority, 11,405 (72.27%), of women, were married, of this 73.3% of women were not use modern contraceptive. In terms of education, 7033 women (44.84%) had no formal education, while 33.24% had an elementary level education. Similarly, 52.27% of their husbands had a secondary or higher degree of education. 42.84% of women were classified as poor, while 39% were classified as wealthy. About 41.47% of women followed as Christian and More than two out of three women, 13,202 (84.1%) had an ANC visit and with 84.087%, of women are not using modern contraceptive method. Only one out of every four women (25.47%) and one out of every four males (26.07%) had heard about family planning in the previous few months. Accordingly, less than 10 percent of women (6.43%) have heard of FP in the last few months from newspapers/magazines. More than half of women (51.27%) were unemployed at the time. Similarly, nearly 90% of the Women partners were currently employed (Table 1).

**Table 1:** Socio-economic, demographic, maternal, and obstetric characteristic of sexual active women in Ethiopia, 2016 Ethiopian Demographic and Health Survey

Variables	Categories	Modern contraceptive, %		Total (*N* = 15,683, %)
		Currently use any modern method (*N* = 3217, 20.51%)	Currently not-use any modern method (*N* = 12,466, 79.49%)	
Region	Tigray	12.25	10.33	1682, 10.72
	Afar	2.52	8.40	1128, 7.19
	Amhara	18.28	9.07	1719, 10.96
	Oromia	12.34	11.99	1892, 12.06
	Somalia	0.50	11.03	1391, 8.87
	Benishangul	7.65	7.06	1126, 7.18
	SNNPR	15.36	10.87	1849, 11.79
	Gambela	6.87	6.53	1035, 6.6
	Harari	5.63	5.82	906, 5.78
	Addis Abeba	12.43	11.42	1824, 11.63
	Dire Dawa	6.19	7.48	1131, 7.21
Residence	Urban	39.26	32.77	5348, 34.10
	Rural	60.74	67.23	10,335, 65.90
Women age (years)	15–24	27.85	44.16	6401, 40.81
	25–34	45.48	29.06	5086, 32.43
	35–49	26.67	26.78	4196, 26.76
Partner age(years)	15–24	2.74	3.49	523, 3.33
	25–34	20.39	20.70	3237, 20.64
	35–59	76.87	75.81	11,923, 76.02
Marital status	Never married	29.59	26.68	4278, 27.28
	Married	70.49	73.32	11,405, 72.27
Women education	No education	41.37	45.74	7033, 44.84
	Primary	35.87	32.56	5213, 33.24
	Secondary and above	22.75	21.70	3437, 21.92
Partner education	No education	25.09	29.07	4431, 28.25
	Primary	18.00	19.85	3054, 19.47
	Secondary and above	56.92	51.07	8198, 52.27
Women occupation	Had work	49.33	48.54	7638,48.70
	Had no work	50.67	51.46	8045, 51.30
Partner occupation	Had work	93.57	93.57	14,675, 93.57
	Had no work	6.43	6.43	1008, 6.43
Wealth index	Poor	30.99	45.89	6718, 42.84
	Middle	23.97	16.66	2848, 18.16
	Rich	45.04	37.45	6117, 39
Religion	Christians	42.28	41.26	6504, 41.47
	Muslim	20.89	17.18	2814, 17.94
	Other	36.84	41.55	6365, 40.59
Births in last five years	No birth	34.35	59.24	8490, 54.17
	1–2	64.13	37.23	6704, 42.75
	3 and more	1.52	3.53	489, 3.12
Health facility delivery	No	25.24	28.74	4395, 28.02
	Yes	74.76	71.26	11,288, 71.98
Accessing Health Care	Big problem	24.90	28.40	4341, 27.68
	No problem	75.10	71.60	11,342, 72.32
Desire for more children	Wants within 2 years	20.21	18.91	3007, 19.17
	Wants after 2 + years	34.88	34.33	5401,34.44
	Unsure timing/ undecided	18.81	19.31	3012, 19.21
	Wants no more/ Sterilized/infecund	26.11	27.46	4263, 27.18
Watched family planning on TV last few months	No	69.26	75.47	11,594, 73.93
	Yes	30.74	24.87	4089, 26.07
Heard family planning on radio last few months	No	70.87	75.47	11,688, 74.53
	Yes	29.13	24.53	3995, 25.47
Read family planning in newspaper/magazine last few months	No	92.14	93.94	14,675, 93.57
	Yes	7.86	6.06	1008, 6.43
ANC visit	No	15.45	15.92	2481, 15.82
	Yes	84.55	84.08	13,202, 84.18

### Multivariable multilevel logistic regression analysis

#### Random effect measures of variation

The results of random effects indicated that there was a statistically significant variation in the non-use of modern contraceptives across the clusters. A two-level mixed-effect binary logistic regression model was used to analyze the effect of women’s individual characteristics and community-level factors in determining women’s non-using of modern contraceptives. Furthermore, the ICC value was 25.41%, indicates that about 25.41% of the total variability of no use of modern contraceptive utilization in Ethiopia were attributed to community-level factors, whereas the individual variation explained the remaining 74.59% of the total variability. After adjusting for individual-level and community-level factors, there is a significant variation in the use of modern contraceptives across communities or clusters.

The proportional change in variance (PCV) in this model was 37.50%, which showed that both community and individual level variables (Table 2) explained 37.50% of community variance observed in the null model. About 37.50% of women with non-use modern contraceptive in clusters were explained in the full model. Besides, MOR was 2.21; it showed that if we randomly select two women from different clusters, a woman from a cluster with high non-use of modern contraceptives was 2.21 times more likely to not to use modern contraceptives than women from the cluster with use of modern contraceptive. This showed that the existence of significant heterogeneity in non-use of modern contraceptives across different communities.

**Table 2:** Multilevel logistic regression analysis of both individual and community-level factors associated with non-contraceptive utilization in Ethiopia, 2016 Ethiopian Demographic and Health Survey

Individual and community level variables	Models			
	Null model	Model I	Model II	Model III
	AOR(95%CI)	AOR(95%CI)	AOR(95%CI)	AOR(95%CI)
Maternal age (years)				
15–24		1		1
25–34		0.50(0.45,0.57)***		0.54(0.47,0.61)***
35–49		0.58(0.51,0.65)***		0.62(0.55,0.71)***
Partner age(years)				
15–24		1		1
25–34		0.75(0.56,0.99)*		0.76(0.57,1.01)
35–59		0.77(0.58,1.02)		0.79(0.61,1.05)
Marital status				
Never married		1		1
Married		1.00(0.87,1.16)		0.99(0.86,1.15)
Maternal education				
No education		1		1
Primary		0.69(0.61,0.78)***		0.77(0.68,0.87)***
Secondary and above		0.71(0.61,0.83)***		0.88(0.75,1.03)***
Partner education				
No education		1		1
Primary		0.91(0.78,1.04)		0.91(0.78,1.04)
Secondary and above		0.84(0.73,0.97)*		0.84(0.72,0.96)**
Maternal occupation				
Had no work		1		1
Had work		1.02(0.93,1.13)		1.03(0.93,1.13)
Partner occupation				
Had no work		1		1
Had work		1.13(0.93,1.38)		1.14(0.94,1.39)
Wealth index				
Poor		1		1
Middle		0.59(0.52,0.69)***		0.66(0.58,0.76)***
Rich		0.68(0.59,0.78)***		0.74(0.65,0.85)***
Religion				
Christians		1		1
Muslim		1.38(1.21,1.64)***		1.43 (1.23,1.62)***
Other		1.01(0.95,1.22)		1.07(0.95,1.22)
Births in last five years				
No birth		1		1
1–2		0.23(0.19,0.24)***		0.21(0.19,0.23)***
3 and more		0.52(0.34,0.67)***		0.37(0.28,0.56)***
Health facility delivery				
No		1		1
Yes		0.85(0.74,0.99)*		0.84(0.73,0.98)*
Accessing Health Care				
Big problem		1		1
No-big problem		0.90(0.81,1.02)		0.91(0.82,1.02)
Desire for more children				
Wants within 2 years		1		1
Wants after 2 + years		1.08(0.95,1.23)		1.09(0.95,1.24)
Unsure timing/ undecided		1.15(0.98,1.35)		1.15(0.98,1.35)
Wants no more/ Sterilized/infecund		1.09(0.95,1.25)		1.11(0.96,1.34)
Watched family planning on TV last few months				
No		1		1
Yes		0.75(0.66,0.86)***		0.74(0.65,0.85)***
Heard family planning on radio last few months				
No		1		1
Yes		0.89(0.79,1.01)		0.91(0.80,1.02)
Read family planning in newspaper/magazine last few months				
No		1		1
Yes		0.91(0.75,1.09)		0.89(0.75,1.09)
ANC visit				
No		1		1
Yes		1.24(1.05,1.46)*		1.25(1.07,1.47)*
Community level variable				
Region				
Tigray			1	1
Afar			5.56(3.73,8.30)***	6.75(4.28,10.66)***
Amhara			0.53(0.39,0.70)***	0.48(0.38,0.67)***
Oromia			1.11(0.83,1.49)	1.32(0.94,1.83)
Somalia			34.85(19.37,62.71)***	47.24(24.99,88.33)***
Benishangul			1.12(0.80,1.55)	1.12(0.0.77,1.63)
SNNPR			0.78(0.58,1.05)	0.88(0.63,1.23)
Gambela			1.53(1.08,2.15)*	1.85(1.25,2.73)*
Harari			1.75(1.23,2.49)**	2.26(1.50,3.39)***
Addis Ababa			1.98(1.41,2.78)***	2.13(1.44,3.15)***
Dire Dawa			2.28(1.59,3.26)***	2.67(1.8,4.02)***
Residence				
Urban			1	1
Rural			2.27(1.87,2.77)***	3.43(2.72,4.32)***
Random effects				
Community variance(SE)	1.12(0.103)	1.44(0.14)	0.49(0.05)	0.69(0.07)
ICC (%)	25.41	30.47	13.15	17.38
PVC (%)	1.00	-28.56	55.36	37.50
MOR	2.73	3.13	1.96	2.21
-2*LL(DIC)	14,972.34	13,461.57	14,517.62	13,003.49
AIC	14,976.34	13,517.57	14,543.62	13,081.49
BIC	14,991.67	13,732.06	14,643.20	13,380.25

NB:* = significant at *P*-value < 0.05;** = significant at *P*-value < 0.01; *** = significant at *P*-value < 0.001

AIC, BIC, and deviance were checked (Table 2), and the multilevel logistic regression model III was chosen because of the smallest value of AIC, BIC, and smallest deviance since the models were nested.

### Individual and community level factors associated with non- use of modern contraceptive utilization among sexually active women in Ethiopia

Table 2 shows the adjusted odds ratios (AOR) derived from a multivariable multilevel logistic regression assessing the likelihood of contraception non-use. Thus, in the multilevel multivariable analysis, maternal age, maternal education, husband education, wealth index, religion, birth in the previous five years, health facility delivery, hearing about FP on TV, Antenatal care utilization, region, and place of residence were significant predictors of non-use of modern contraception in Ethiopia. Women aged 34–49 years (AOR = 0.54, 95% CI: 0.5–0.61) and age between 34–49 years (AOR = 0.62, 95% CI: 0.55–0.71) were less likely to not utilize contraception than women aged 15–24 years. When compared to those who did not have any formal education, those who attended primary school (AOR = 0.77, 95% CI: 0.68–0.87) and secondary and above educational level (AOR = 0.88, CI: 0.75–1.03) were less likely to not use contraception. Similarly, husbands with secondary and above educational level (AOR = 0.83, 95% CI: 0.72–0.96) had a higher likelihood of being non-users of contraceptives compared to their reference group. As compared to women from poor households, women from middle (AOR = 0.66, 95% CI: 0.58–0.76) and rich (AOR = 0.74, 95% CI: 0.65–0.85) wealth level had lower probabilities of not using contraception. Relative to Orthodox Christian respondents, Muslim respondents (AOR = 1.43, 95% CI: 1.23–1.62) were more likely to not use contraception. The odds of not using contraception were lower among women who had 1–2 births (AOR = 0.21, 95% CI: 0.19–0.23) and 3 or more births (AOR = 0.37, 95% CI: 0.28–0.56) compared to women who had no birth. Women who gave birth in a health facility (AOR = 0.84, 95%CI: 0.73–0.0.98) had a lower chance of not using contraception than women who gave birth at home. Women who watched family planning information on TV were less likely to not use contraception (AOR = 0.74, 95% CI: 0.65–0.85). Women living in Afar (AOR = 6.75: 95%CI: 4.28–10.66), Somali (AOR = 47; 95% CI: 24.9–88.9), Gambela (AOR = 1.85:95% CI:1.25–2.73), Harari (AOR = 2.25; 95%CI: 1.50–3.39), Addis Ababa (AOR = 2.13:95% CI:1.44–3.15) and Dire Dawa region (AOR = 2.67; 95%CI: 1.8–4.02) were more likely to not use contraception as compared to women living in Tigray region. However, Women living in Amhara (AOR = 0.48; 95%CI: 0.38–0.67) were less likely to not use modern contraceptive as compared to women living in the Tigray region. Rural women (AOR = 3.43; 95% CI: 2.72–4.32) had a higher odds of not using contraception (AOR = 3.43; 95 percent CI: 2.72–4.32) than urban women (Table 2).

## Discussion

The ability of girls and women to control their fertility, to choose whether and when to have children, and how many children to have, is at the heart of women’s empowerment, gender equality, and progress for all. As a result, the aim of this study was to determine the prevalence of non-use of modern contraceptive method and associated factors among Ethiopian women of reproductive age.

The findings of this study revealed that 79.49% of sexually active women did not use a modern contraceptive method, which is consistent with findings from studies in Ethiopia (79.5%) [20, 25] and Ghana (78.5%) [34]. Furthermore, as women’s ages increased, their use of FP decreased. The women’s age had a significant impact on their refusal to use modern contraceptive methods. This finding was consistent with the findings of a Nepal [35, 36], and Malawi [37] study, which discovered that as women’s ages increase, so does their likelihood of using modern contraception. Studies conducted in China [34], and Ethiopia [20, 35, 38] found results that differed from this one. The low contraceptive prevalence among women aged 15–24 years is most likely due to the fact that the majority of these women engage in unsafe sex, are newly married, and marriage is based on the institution of producing children. Access to modern FP services is likely to be difficult for a young mother. Another possible explanation is that the former group included women who had either finished child bearing or wanted to space their children, as opposed to the latter group, which included women who had not yet begun child bearing. Additional possible reason could be that youths were less likely to use family planning services because of societal beliefs that they should not have premarital sex.

This study revealed that an increasing educational level of women were more likely to use a modern contraceptive method. This suggests that women education most likely has a positive effect on use of modern FP. This result was consistent with previous studies [26, 37, 39, 40]. This could be explained by the fact that educated women have better access to health care information, have greater autonomy to make decisions, and have a greater ability to use quality health care services. Moreover, educated individuals might be busy by the nature of their work and have no time to take care of their child, and they plan to use contraceptive methods to decrease the burden of being pregnant and child care [41–45].

There is a significant association between women wealth status and non-use of modern contraceptive method. The result of the current study was in line with the studies conducted in Nepal [36], Ethiopia [20, 35, 38], Rwanda [46], Burkina Faso [47], and Nigeria [48].This might be due to the fact that women from rich households might be more educated and have occupations, as supported by this study, to extend their business agendas and further. When women’s occupation status increases, they will be more inclined to use contraception because their support gives them more control over reproductive health and the desire to limit family size [49]. Moreover, in our community, most rich women had one or two children throughout their lifetime, and this indicates that they are more likely to utilize modern contraceptive methods [35].

Religion, according to the study's findings, is strongly associated with women’s non-use of modern contraception. Muslims women were more likely to not use contraception compared Christian women. This finding is consistent with the findings of [20, 36, 50]. This could be due to the Muslim community's strong belief in a holy book that prohibits family planning [34]. This result's attribute necessitates additional research.

Women, who had given birth to a child in the last 5 years were less likely to non-use modern contraceptive than those women who had not given birth. This result is in agreement with other studies [20, 35, 36, 51]. The odds of not use modern contraceptive method among women delivered at the health facility were lower by 0.84 times as compared to women deliver at home. The attitude of non-use of modern contraception methods appeared to be influenced by exposure to mass media such as radio, TV, and newspapers. This could be because women who have had media exposure are more aware of modern contraceptives and how to use them. Our findings show a link between exposure to mass media such as radio, television, and newspapers and contraceptive non-use. In the Nepal [36] and Ethiopia [10, 20, 26], a similar result was seen. This finding was backed up by studies conducted in Nepal [36], BurkinaFaso [47], Ethiopia [20, 47, 52], and Nigeria [48, 53].The possible explanation is that women who give birth in a health facility may receive guidance and counseling about the benefits of modern contraceptive use from health professionals, and the practice of individual health education has slightly increased the uptake of FP methods. As a result, mass media probably appropriate for disseminating information, increasing awareness and encouraging women to use modern contraception.

Women in urban may have more confidence in their decision-making abilities, autonomy, access to contraception methods, and even higher living standards than women in rural areas [51]. Studies conducted in Ethiopia [20, 54], Nepal [36], and Nigeria [53], respectively, have shown that women who live in rural areas were more likely to not use modern contraceptives method than those who live in urban areas. This suggests that other factors that promote contraceptive use and are more prevalent in urban areas exist. These include the education and wealth of women. Moreover, the non-use of modern contraception methods varied by region. Women in Afar, Somali, Gambela, Harari, and Dire Dawa were less likely to use modern contraception methods than women in Tigray, but Amhara region had a lower rate of non-use. This result was confirmed the studies.The possible reason for this regional disparity is that there are differences in the implementation of family planning services across regions. Contraceptive methods are inaccessible, resulting in Ethiopia's highest under-five mortality rate [20, 50, 54, 55]. This implies that having access to contraceptive methods will reduce child and infant mortality and add to the health complications of mothers.

## Conclusion

In the current study, the magnitude of non-use of modern contraceptive utilization among sexually active women in Ethiopia is unexpectedly high. Among individual-level factors, aged women, educated women, educated husbands, women who had at least one birth in the previous five years, hospital delivery, and watching TV were negatively associated with non-use of modern contraceptive, but poor women, Muslim women, and having ANC visit were positively associated with non-use of contraceptive. In Ethiopia, community-level factors such as place of residence and region were significantly associated with non-use of modern contraceptives. As a result, the government and other stakeholders must provide educational opportunities, raise awareness about the use of modern contraceptives, and provide valuable counseling services to those who may be avoiding modern contraceptive methods.

## Data Availability

The survey datasets used in this study was based on a publicly available data set that is freely available online with no participant’s identity from http://www.dhsprogram.com/data/available-datasets.cfm. Approval was sought from MEASURE DHS/ICF International and permission was granted for its use.

## Abbreviations

AIC: Akaike’s information criterion
AOR: Adjusted odds ratio
CI: Confidence intervals
DIC: Deviance information criterion
EAs: Enumeration areas
EDHS: Ethiopian demographic and health survey
FP: Family Planning
ICC: Intra-cluster correlation
MOR: Median odds ratio
PCV: Proportional change in variance
SNNPR: Southern Nations, Nationalities, and People Region

## References

[1] Ahinkorah BO, Budu E, Aboagye RG, Agbaglo E, Arthur-Holmes F, Adu C (2021). Factors associated with modern contraceptive use among women with no fertility intention in sub-Saharan Africa: evidence from cross-sectional surveys of 29 countries. Contracept Reprod Med.

[2] World Health Organization (2014). Sucess factors for women’s and children’s health: policy and programme highlights from 10 fast-track countries.

[3] World Health Organization (2020). HRP annual report 2019.

[4] Engelbert Bain L, Amu H, Enowbeyang TE (2021). Barriers and motivators of contraceptive use among young people in Sub-Saharan Africa: A systematic review of qualitative studies. PLoS ONE.

[5] Sserwanja Q, Musaba MW, Mukunya D (2021). Prevalence and factors associated with modern contraceptives utilization among female adolescents in Uganda. BMC Womens Health.

[6] Gribble J, Haffey J. Reproductive health in sub-Saharan Africa. Population reference bureau. 2008;8.

[7] Levandowski BA, Kalilani-Phiri L, Kachale F, Awah P, Kangaude G, Mhango C (2012). Investigating social consequences of unwanted pregnancy and unsafe abortion in Malawi: the role of stigma. Int J Gynecol Obstet.

[8] Ganatra B, Faundes A (2016). Role of birth spacing, family planning services, safe abortion services and post-abortion care in reducing maternal mortality. Best Pract Res Clin Obstet Gynaecol.

[9] Stover J, Winfrey W (2017). The effects of family planning and other factors on fertility, abortion, miscarriage, and stillbirths in the Spectrum model. BMC Public Health.

[10] Tsui AO, McDonald-Mosley R, Burke AE (2010). Family planning and the burden of unintended pregnancies. Epidemiol Rev.

[11] Cleland J, Bernstein S, Ezeh A, Faundes A, Glasier A, Innis J (2006). Family planning: the unfinished agenda. Lancet.

[12] Csa I. Central Statistical Agency (CSA)[Ethiopia] and ICF. Ethiopia Demographic and Health Survey, Addis Ababa. Ethiopia and Calverton. 2016.

[13] World Health Organization. Trends in maternal mortality 2000 to 2017: estimates by WHO, UNICEF, UNFPA, World Bank Group and the United Nations Population Division. 2019;

[14] Gebremedhin AY, Kebede Y, Gelagay AA, Habitu YA (2018). Family planning use and its associated factors among women in the extended postpartum period in Addis Ababa. Ethiopia Contracept Reprod Med.

[15] Jima GH, Nemo H (2020). Postpartum Family Planning Utilization and associated factors among Women who gave birth in the last 12 months in selected Districts of Arsi Zone, Southeast Ethiopia: A community based cross-sectional study. Arsi Journal of Science and Innovation.

[16] Assefa Y, Van Damme W, Williams OD, Hill PS (2017). Successes and challenges of the millennium development goals in Ethiopia: lessons for the sustainable development goals. BMJ Glob Heal.

[17] Tafere TE, Afework MF, Yalew AW (2018). Counseling on family planning during ANC service increases the likelihood of postpartum family planning use in Bahir Dar City Administration, Northwest Ethiopia: a prospective follow up study. Contracept Reprod Med.

[18] Teka TT, Feyissa TR, Melka AS, Bobo FT (2018). Role of antenatal and postnatal care in contraceptive use during postpartum period in western Ethiopia: a cross sectional study. BMC Res Notes.

[19] Endriyas M, Eshete A, Mekonnen E, Misganaw T, Shiferaw M, Ayele S (2017). Contraceptive utilization and associated factors among women of reproductive age group in Southern Nations Nationalities and Peoples’ Region, Ethiopia: cross-sectional survey, mixed-methods. Contracept Reprod Med.

[20] Fenta SM, Gebremichael SG (2021). Predictors of modern contraceptive usage among sexually active rural women in Ethiopia: A multi-level analysis. Arch Public Heal.

[21] Tadesse M, Teklie H, Yazew G, Gebreselassie T. Women’s empowerment as a determinant of contraceptive use in Ethiopia further analysis of the 2011 Ethiopia demographic and health survey. DHS Further Analysis Reports. 2013:82.

[22] Tessema GA, Mekonnen TT, Mengesha ZB, Tumlinson K (2018). Association between skilled maternal healthcare and postpartum contraceptive use in Ethiopia. BMC Pregnancy Childbirth.

[23] Teferra AS, Wondifraw AA. Determinants of long acting contraceptive use among reproductive age women in Ethiopia: evidence from EDHS 2011.

[24] Gordon C, Sabates R, Bond R, Wubshet T (2011). Women’s education and modern contraceptive use in Ethiopia. Int J Educ.

[25] Gebre MN, Edossa ZK (2020). Modern contraceptive utilization and associated factors among reproductive-age women in Ethiopia: evidence from 2016 Ethiopia demographic and health survey. BMC Womens Health.

[26] Agbadi P, Tagoe E, Akosua AF, Owusu S. A multilevel analysis of predictors of modern contraceptive use among reproductive age women in Sierra Leone: insight from demographic and Health surveys. Center for Open Science. 2019.

[27] Idris H (2019). Factors Affecting the Use of Contraceptive in Indonesia: Analysis from the National Socioeconomic Survey (Susenas). KEMAS.

[28] Merlo J (2014). Invited commentary: multilevel analysis of individual heterogeneity—a fundamental critique of the current probabilistic risk factor epidemiology. Am J Epidemiol.

[29] Austin PC, Stryhn H, Leckie G, Merlo J (2018). Measures of clustering and heterogeneity in multilevel P oisson regression analyses of rates/count data. Stat Med.

[30] Zuur AF, Ieno EN, Walker NJ, Saveliev AA, Smith GM (2009). Mixed effects models and extensions in ecology with R.

[31] Halonen JI, Kivimäki M, Pentti J, Kawachi I, Virtanen M, Martikainen P (2012). Quantifying neighbourhood socioeconomic effects in clustering of behaviour-related risk factors: a multilevel analysis. PLoS ONE.

[32] Katz MH. Multivariable analysis: a practical guide for clinicians and public health researchers. Cambridge university press; 2011.

[33] Multicollinearity AA (2010). Wiley Interdiscip Rev. Comput Stat.

[34] Alomair N, Alageel S, Davies N, Bailey JV (2020). Factors influencing sexual and reproductive health of Muslim women: a systematic review. Reprod Health.

[35] Tessema ZT, Teshale AB, Tesema GA, Yeshaw Y, Worku MG (2021). Pooled prevalence and determinants of modern contraceptive utilization in East Africa: A Multi-country Analysis of recent Demographic and Health Surveys. PLoS ONE.

[36] Adhikari R, Acharya D, Ranabhat CL, Ranju KC (2019). Factors associated with non-use of contraceptives among married women in Nepal. J Heal Promot.

[37] Mandiwa C, Namondwe B, Makwinja A, Zamawe C (2018). Factors associated with contraceptive use among young women in Malawi: analysis of the 2015–16 Malawi demographic and health survey data. Contracept Reprod Med.

[38] Tegegne TK, Chojenta C, Forder PM, Getachew T, Smith R, Loxton D (2020). Spatial variations and associated factors of modern contraceptive use in Ethiopia: a spatial and multilevel analysis. BMJ Open.

[39] Debebe Z, Tachbele E, Dereje N. Predictors of modern contraceptive utilization among married reproductive age women in Misha district, Southern Ethiopia: A community based cross sectional study.

[40] Idris H (2019). Factors Affecting the Use of Contraceptive in Indonesia: Analysis from the National Socioeconomic Survey (Susenas). KEMAS J Kesehat Masy.

[41] Pazol K, Zapata LB, Tregear SJ, Mautone-Smith N, Gavin LE (2015). Impact of contraceptive education on contraceptive knowledge and decision making: a systematic review. Am J Prev Med.

[42] Lopez LM, Grey TW, Hiller JE, Chen M. Education for contraceptive use by women after childbirth. Cochrane Database of Systematic Reviews. 2015;(7). 10.1002/14651858.CD001863.pub4 PMC7154222 26222129

[43] Buyinza F, Hisali E (2014). Microeffects of women’s education on contraceptive use and fertility: The case of Uganda. J Int Dev.

[44] Koch E, Calhoun B, Aracena P, Gatica S, Bravo M (2014). Women’s education level, contraceptive use and maternal mortality estimates. Public Health.

[45] Gordon C, Sabates R, Bond R, Wubshet T (2011). Women’s Education and Modern Contraceptive Use in Ethiopia. Int J Educ.

[46] Muhoza ND (2014). Excess Fertility and Family Planning in Rwanda: Understanding the shift to a high contraceptive prevalence country.

[47] Hounton S, Barros AJD, Amouzou A, Shiferaw S, Maïga A, Akinyemi A (2015). Patterns and trends of contraceptive use among sexually active adolescents in Burkina Faso, Ethiopia, and Nigeria: evidence from cross-sectional studies. Glob Health Action.

[48] Ejembi CL, Dahiru T, Aliyu AA. Contextual factors influencing modern contraceptive use in Nigeria. DHS Work Pap. 2015;(120).

[49] Islam MS, Alam MS, Hasan MM (2014). Inter-spousal communication on family planning and its effect on contraceptive use and method choice in Bangladesh. Asian Soc Sci.

[50] Walelign D, Mekonen A, Netsere M, Tarekegn M (2014). Modern contraceptive use among orthodox Christian and Muslim women of reproductive age group in Bahir Dar city, north West Ethiopia: comparative cross sectional study. Open J Epidemiol.

[51] Abate MG, Tareke AA (2019). Individual and community level associates of contraceptive use in Ethiopia: a multilevel mixed effects analysis. Arch Public Heal.

[52] Asresie MB, Fekadu GA, Dagnew GW (2020). Contraceptive use among women with no fertility intention in Ethiopia. PLoS ONE.

[53] Asaolu I, Nuño VL, Ernst K, Taren D, Ehiri J (2019). Healthcare system indicators associated with modern contraceptive use in Ghana, Kenya, and Nigeria: evidence from the Performance Monitoring and Accountability 2020 data. Reprod Health.

[54] Tegegne TK, Chojenta C, Forder PM, Getachew T, Smith R, Loxton D (2020). Spatial variations and associated factors of modern contraceptive use in Ethiopia: a spatial and multilevel analysis. BMJ Open.

[55] Seyife A, Fisseha G, Yebyo H, Gidey G, Gerensea H (2019). Utilization of modern contraceptives and predictors among women in Shimelba refugee camp, Northern Ethiopia. PLoS ONE.

